# Perspectives on (A)symmetry of Arcuate Fasciculus. A Short Review About Anatomy, Tractography and TMS for Arcuate Fasciculus Reconstruction in Planning Surgery for Gliomas in Language Areas

**DOI:** 10.3389/fneur.2021.639822

**Published:** 2021-02-10

**Authors:** Andrea Di Cristofori, Gianpaolo Basso, Camilla de Laurentis, Ilaria Mauri, Martina Andrea Sirtori, Carlo Ferrarese, Valeria Isella, Carlo Giussani

**Affiliations:** ^1^Neurosurgery Unit, San Gerardo Hospital, ASST Monza, Monza, Italy; ^2^School of Medicine and Surgery, University of Milano-Bicocca, Milan, Italy; ^3^Neuroradiology Unit, San Gerardo Hospital, ASST Monza, Monza, Italy; ^4^Neurology Unit, San Gerardo Hospital, ASST Monza, Monza, Italy; ^5^Department of Psychology, University of Milano-Bicocca, Milan, Italy

**Keywords:** glioma, surgery, planning, arcuate fasciculus, tranancranial magnetic stimulation, diffusion tensor imaging, white matter asymmetries, white matter anatomy

## Abstract

Gliomas are brain tumors that are treated with surgical resection. Prognosis is influenced by the extent of resection and postoperative neurological status. As consequence, given the extreme interindividual and interhemispheric variability of subcortical white matter (WM) surgical planning requires to be patient's tailored. According to the “connectionist model,” there is a huge variability among both cortical areas and subcortical WM in all human beings, and it is known that brain is able to reorganize itself and to adapt to WM lesions. Brain magnetic resonance imaging diffusion tensor imaging (DTI) tractography allows visualization of WM bundles. Nowadays DTI tractography is widely available in the clinical setting for presurgical planning. Arcuate fasciculus (AF) is a long WM bundle that connects the Broca's and Wernicke's regions with a complex anatomical architecture and important role in language functions. Thus, its preservation is important for the postoperative outcome, and DTI tractography is usually performed for planning surgery within the language-dominant hemisphere. High variability among individuals and an asymmetrical pattern has been reported for this WM bundle. However, the functional relevance of AF in the contralateral non-dominant hemisphere in case of tumoral or surgical lesion of the language-dominant AF is unclear. This review focuses on AF anatomy with special attention to its asymmetry in both normal and pathological conditions and how it may be explored with preoperative tools for planning surgery on gliomas in language areas. Based on the findings available in literature, we finally speculate about the potential role of preoperative evaluation of the WM contralateral to the surgical site.

## Introduction

Gliomas are intra-axial infiltrating brain tumors, boundaries of which within the perilesional white matter (WM) are very difficult to define (1, 2). It is accepted that the best prognosis can be reached aiming at the most radical tumor resection possible preserving, however, a good postoperative neurological status (3, 4). In fact, The poor quality of life of patients with postoperative deficits leads to an overall survival reduction (5–8). As a consequence, the extent of resection within the possibly infiltrated but normal-appearing brain tissue must avoid damaging critical normal functioning cortical areas and their WM connections (9–11). Within the neurosurgical planning, the definition of what brain tissue must be considered “eloquent” is still mostly based on a classic localizationist model, which assumes that cortical areas are specialized for specific aspects of neurological functions. On the other hand, this localizationist model is now outdated, and cognitive neuroscience research suggests that every cognitive and motor function depends on complex neuroplastic networks connecting many cortical areas by means of long- and short-association WM fibers (12, 13). According to this connectionist model, cortical, and subcortical functional interplay may be highly variable among human beings and can reorganize in response to brain damage, such as an acute brain insult or a relatively slow-growing tumor mass (10, 14–17). These so-called neuroplastic properties might explain why, despite bearing an extensive mass located close or within brain tissue considered to be critical according to the classic localizationist model, patients with intra-axial brain tumors usually display very mild or even no neurological deficit at presentation and can maintain this status even after surgery with extensive tumoral and peritumoral tissue resection (14, 17–19). Thus, the concept of neuroplasticity should be considered for accurate presurgical planning, but full translation of this factor from basic to clinical neuroscience is still lacking because of incomplete understanding of its mechanisms. In fact, currently, it is still impossible to predict its impact on the postsurgical outcome based on an objective measure of how much the brain of the patient has already reorganized in response to the tumor and may still reorganize after surgery (20, 21). From this point of view, cortical and subcortical structures show different plastic potential. In fact, cortex seems to have a great neuroplastic potential, and it is able to reorganize effectively in case of brain tumors, while WM bundles seem to have a low plastic potential (22, 23), and extensive surgical resections might depend mainly on WM boundaries rather than on the cortical extension of the tumor (24–26). One of the most critical functions to be preserved after surgery is verbal language. Both neuropsychological studies in patient and functional studies in neurologically normal subjects have clarified that language functions are strongly lateralized to one hemisphere, defined as dominant for language (27–29). The network of areas involved comprises cortical regions that are present and overall symmetrical within the two hemispheres. However, only few individuals have a right-hemisphere dominance, which ranges from 4% in strong right-handers to 15% in ambidextrous to 27% in strong left-handers (30).

The strong well-known cortical lateralization of language functions is one of the key factors for preoperative brain tumor risk assessment in adults. A low risk is usually assigned if the lesion is located far from areas involved in this function, with the lowest risk associated with lesions located within the non-dominant hemisphere (29, 31). However, it is known that a brain lesion to the dominant hemisphere may induce a complex cortical functional reorganization of the language network that may involve a complex interplay of activity with contralateral homologous cortical areas and functional reorganization in the perilesional cortex, while it is still unclear how WM can functionally accommodate a brain damage induced by the tumor given its low neuroplastic potential (21, 22). This prevents *a priori* assumption of language lateralization in a patient with a brain tumor. Wada test was the first reliable method developed to establish the dominant hemisphere for language. Subsequently, the advances of functional magnetic resonance imaging (MRI) studies, with which an indirect measure of brain activity can be assessed based on blood oxygen level modifications [functional MRI (fMRI)], proved to be a reliable non-invasive and riskless substitute of the Wada test. Thus, fMRI is currently used to define hemispheric dominance, computing a laterality index based on the comparison of relative activation of right and left frontal and temporal areas induced by language tasks execution (32–34). Nevertheless, language functions involve activity of a vast network of areas, spanning all lobes of the dominant hemisphere. Thus, the functional properties of this network strongly depend on intrinsic efficient connections among them, and presurgical assessment must take into account also the risk of damaging critical WM bundles (31, 35, 36).

Magnetic resonance multidirectional diffusion-weighted imaging (MD-DWI) tractography allows *in vivo* indirect reconstruction of WM fibers and can be used to perform digital anatomical dissections of WM bundles (37–39). Many different MD-DWI tractography acquisition protocols, postprocessing methods, and reconstruction algorithms are currently used with different accuracy levels, and all of them are prone to false positive and false negative (40). The simplest MD-DWI tractography method is based on deterministic tracing based on voxel main diffusion direction estimated with tensor modeling [diffusion tensor imaging (DTI)]. DTI tractography (DTI-T) is currently used for preoperative surgical planning to maximize the surgical resection avoiding to damage association and projection fibers located nearby the tumor (6, 35, 41) and consistently reveals asymmetries of WM tracts between the two hemispheres (37, 42) that may be paired with cortical lateralization of cognitive and motor functions. Nevertheless, to our knowledge, very few studies explored the clinical relevance of these structural asymmetries for the functional outcome after brain surgery for gliomas. Moreover, it is still unclear if subcortical variability could have a role in postoperative outcome and if it could be of some utility for the neurosurgeon to predict the risk of neurological deficits (9, 35). In this mini-review, we focus on available evidence in the literature about one bundle considered of paramount importance for language functions, the arcuate fasciculus (AF), to better understand the meaning of its asymmetry for preoperative risk assessment of patients undergoing surgical resection for gliomas.

## Arcuate Fasciculus Anatomy and Functional Relevance for Language Within The Dominant Hemisphere

The AF has been involved in the neurobiology of language since Geschwind's (43) proposal of the “classic model,” where AF was the only link between two broad anatomical regions, Broca's territory in the inferior frontal and precentral gyri and Wernicke's territory in the posterior temporal lobe, functionally specialized, respectively, for language production and language comprehension. During the last three decades, the field of language neurobiology has been slowly but steadily moving toward the definitive overcoming of the classic model (44–47). The “dual stream” model proposed by Hickok and Poeppel (48) was a major step forward both because it expanded the number of cortical regions contributing to language functions and suggested that, as previously proposed for the visuospatial system (49), some language abilities may depend more on the functional interplay among them more than on their cortical functional specialization. A critical role of AF was maintained also within this model as revealed by direct electrical stimulation of WM bundles during neurosurgery (23, 50), suggesting that the AF may be the critical anatomical connection within the dorsal functional stream devoted to speech articulation and excluding any of its role for speech comprehension that appeared to be supported by a ventral stream. Yet, even the dual-stream model does not fully explain the spectrum of aphasia symptoms resulting from ischemic brain damage and has been recently revised (46).

The work of Hickok and Poeppel, however, started a cortical delocalization conceptual shift that is leading to the new theoretical framework of a “language connectome” within which language functions would emerge from the dynamic of connections of many cortical and subcortical regions, which may have no specific language properties itself (25, 45–47). Within this view, WM bundle exploration has gained more and more importance as they provide the fundamental anatomical support for the correct functioning of the connectome. So far, many sets of intralobe, intrahemispheric, and interhemisheric association WM bundles connecting frontal, temporal, parietal, insular, and occipital areas have been identified as possibly supporting language functions (25, 45, 47). Among them, probably AF still remains the most studied WM bundle since its very first dissection description by Reil (51). Nevertheless, AF anatomy has been and is still matter of debate (39, 45, 52–54).

A simple virtual dissection method to identify AF with DTI-T has been proposed by Catani (12), and we currently use this method routinely in our glioma presurgical settings. It allows separating the bulk of fibers passing through the perisylvian lateral frontoparietal-temporal WM into three subsets. Two lateral short segments connect the inferior parietal lobule (IPL) with both the frontal operculum and the middle and superior temporal gyrus (MTG/STG), respectively, through an anterior and horizontal bundle and a posterior vertical one. The third medial segment corresponds to the proper AF, which directly connects a wide frontal cortical area made of the frontal operculum, the middle frontal gyrus, and the inferior precentral gyrus, to a wide temporal area comprising the posterior portion of MTG and STG, even if this last terminations are highly variable, probably due to intrinsic limitations of DTI-T (55). A more detailed definition of AF can be reached with high-resolution MD-DWI tractography. Using one of these methods, Fernandez-Miranda et al. (39) conducted a tractography study on 10 healthy subjects and found that STG, MTG, and ITG contributed equally to the AF with a strong left lateralization for STG and ITG, whereas MTG contributed equally in left and right AF. Concerning the frontal counterpart, AF fibers terminated in the pars opercularis in all subjects in the left part, but only in 3 subjects in the right hemisphere; the pars triangularis was a termination site in 3/10 subjects in the left and in 5/10 subjects in the right hemisphere; the ventral precentral gyrus was a site of termination of AF fibers in 8/10 subjects and in 2/10 subjects, respectively, in left and right hemispheres (39). Such distribution was similar to the one described by Martino and colleagues in 2013 on WM *postmortem* dissections (55). In 2016, Yagmurlu et al. proposed a different organization of the left-sided AF after *postmortem* WM dissections of 25 brains (54). They described the segmentation of AF as composed of a ventral and dorsal segment, in line with the model of Glasser and Rilling according to whom the AF is divided into two segments, one terminating in STG and another terminating in MTG (56). Despite detailed WM *postmortem* and WM *in vivo* dissections with several tractography techniques, cortical terminations of the AF are still a matter of debate (44, 52), with discrepancies that have been highlighted in a recent review by Bernard et al. (53).

Within the theoretical framework of the language connectome, this uncertainty poses substantial problems in terms of the functional meaning of the AF whose importance for language functions, however, has been consistently proven by intraoperative brain mapping and lesion studies (2, 31, 57, 58).

In 2012, Bizzi et al. described the importance of AF in determining preoperative aphasia. Interestingly, they showed how aphasia in patients with gliomas was related with tumoral damage to the subcortical WM more than to the infiltration of the cortex offering an indirect proof about the difference of cortical and subcortical plasticity (29). In a recent study on 54 patients undergoing surgical resection of a brain tumor, Li and colleagues showed that onset of postoperative aphasia was associated with a resection border distance to AF <5 mm as seen on postoperative DTI-T (59). In 2018, Ille et al. studied 10 patients with preoperative and postoperative DTI-T for surgical planning of left-sided glioma resection. They found that integrity of AF at DTI-T correlated with preservation of language functions, whereas patients who showed postoperative DTI-T loss of AF fibers manifested a non-fluent aphasia (60). Results by Ille et al. and Li et al. are in line with previous findings by Caverzasi et al. (61). They described preoperative and postoperative diffusion tractography of 78 patients harboring a glioma in the left hemisphere and undergoing surgical resection and found that preservation of AF was associated with a better outcome in terms of language function also in those patients with an early postoperative speech deficit (61).

Taken together, these studies demonstrate that damage to AF within the dominant hemisphere is a crucial factor for the onset of aphasia. Moreover, a damage of AF is also suspected to hinder the reorganization of subcortical components of the entire language network that would be necessary for recovery from aphasia. Recently, in fact, it has been proposed that the dominant IFOF and AF should be considered as “non-resectable” tracts in contrast to other WM tract that can be resected without inducing deficits (50). For example, Inferior Longitudinal Fasciculus (ILF) can be considered “resectable” with a high compensatory index, whereas Inferior Fronto-Occipital Fasciculus (IFOF) can be considered with a low plastic potential when damaged in its middle and posterior part (22). AF is known to have a low compensatory index with a low plastic potential (10). AF and IFOF bear a rich bulk of connections between the temporal and frontal lobes and subserve a wide range of cognitive functions. The neuroplasticity potential of subcortical tracts might not be sufficiently efficient for a rewiring of such widespread connectivity in the presence of damage to the IFOF and AF (20). To this respect, AF representation within the non-dominant hemisphere may be of some relevance.

## Lateralization of Arcuate Fasciculus in Relation To Hemispheric Language Dominance and Recovery From Aphasia

WM interindividual variability and interhemispheric variability have been confirmed by Rademacher in 2001 and by Bürgel et al. in *postmortem* studies with microarchitectonic and MRI dissections (62, 63). In particular, WM symmetry was found on fiber tracts undergoing early myelinization like the corticospinal tract and the optic radiation, while an important asymmetry among long tracts such as AF was found, which undergo a later myelinization during the ontogeny (64). More specifically, asymmetry of the AF has been repeatedly reported in the normal brain. The direct segment, in particular, seems to be often undetected in one of the two hemispheres, usually the non-dominant one (37, 42). Such asymmetry is revealed also by DTI-T studies. Catani and colleagues found that in 62.5% of a group of healthy right-handers the AF could be reconstructed only on the left side, whereas in another 20% of subjects, it was bilateral but showed a significant leftward asymmetry (42). Such results have been confirmed by high-resolution diffusion tractography (39), but some contradicting results are reported in few WM dissection studies (37, 54, 55). Despite these discrepancies, the asymmetrical representation of AF is overall a robust finding to justify the question about its relevance for language lateralization and its possible role for aphasia recovery, as suggested by some studies that have started exploring this issue using diffusion tractography (15, 19, 65).

Forkel et al. (15) suggested that the degree of recovery from aphasia after brain ischemia may be significantly related with the volume of the right AF, suggesting a possible compensative role of AF within the non-dominant hemisphere in case of subcortical damage of the dominant one. This is particularly true in children, as demonstrated by Goradia et al. on 10 children operated on for left temporal lobe epilepsy (65). They documented an increase of the fiber density in right AF of 8 of 10 patients as a compensatory mechanism after surgery, with only one patient experiencing a decline in language performance after surgery (65). A recent study by Jiao et al. conducted on patients undergoing surgical removal of brain arteriovenous malformations in the left IPL confirmed the compensatory role of the non-dominant AF (16). In all patients of this study, surgery was associated with a damage of the left AF, which resulted in language deficit. However, DTI-T performed 6 months after surgery documented an increased number of reconstructed fibers of the right AF, and 5 of 6 patients recovered from the postoperative language deficit with a reorganization of their language areas in the right hemisphere as revealed by fMRI data (16). Similar results are reported in a case described by Chernoff (66). Taken together, these findings might suggest that the right AF may sustain functional recovery from aphasia and reorganization of the language brain network even after surgical damage.

**Figure 1:**
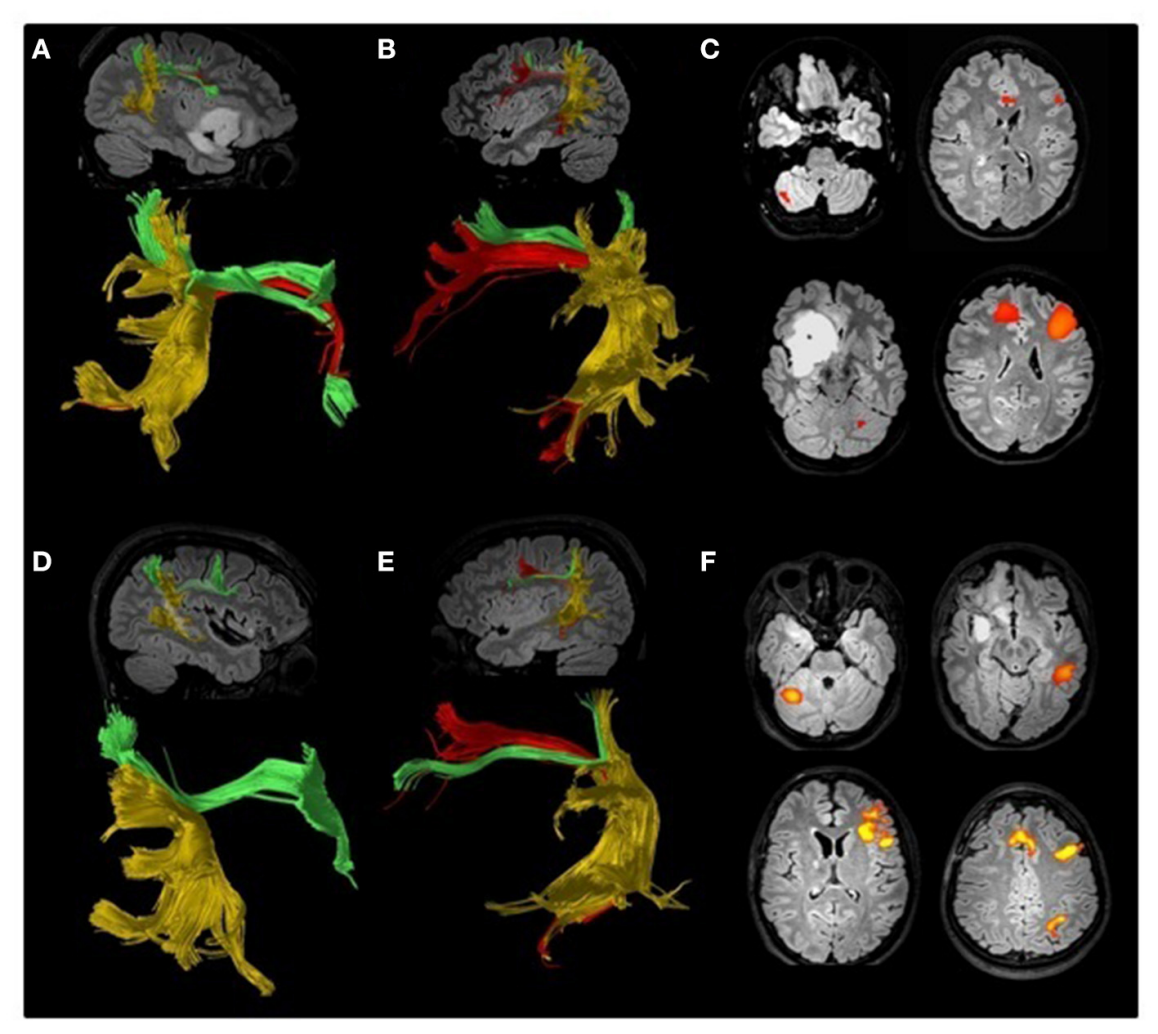
Preoperative **(A–C)** and postoperative **(D–F)** DTI tractography and fMRI study of an 18-year-old ambidextrous woman with a right frontotemporoinsular low-grade glioma. Arcuate fasciculus (AF, red) has been reconstructed according to the method proposed by Catani (12) separating it from the anterior horizontal (green) and posterior vertical (yellow) bundles. A lower number of AF reconstructed fibers can be seen on the right hemisphere **(A)** compared to the left **(B)**. Preoperative fMRI **(C)** suggested left language lateralization. Direct cortical stimulation performed during awake surgery detected episodes of speech arrest, suggesting some degree of language lateralization to the right hemisphere. Postoperative DTI confirmed the asymmetry between the right **(D)** and left **(E)** AF, and fMRI **(F)** was consistent with a clear lateralization of language functions to the left hemisphere.

As it may be expected, asymmetrical representation of AF is commonly found also in DTI-T performed for surgical planning of patients with gliomas. An example of AF asymmetry in one patient treated at our hospital can be seen in Figure 1, whereas Table 1 reports the main studies including AF tractography for surgical planning on patients with gliomas. Only few of them took into account contralesional WM bundles (19, 69, 70). In 2018, Jehna et al. demonstrated that, at presentation, language functions were worse in patients in whom the AF was left-lateralized, whereas patients with symmetric or right-lateralized AF reconstructions showed better language performance (19). Moreover, within their cohort, patients with rapidly growing tumors showed worse performance at verbal semantic fluency test at presentation when compared with slow-growing tumor patients. Similar findings were reported by Incekara et al. (68). They found that patients in which gliomas were associated with microstructural changes of the AF had a low verbal semantic fluency, and those harboring a high-grade glioma had the worst performance (68). These few studies suggest that contralateral AF may play a role also in adults harboring a glioma within the language-dominant hemisphere.

**Table 1:** The main articles cited in this mini review and focused on presurgical planning, DTI-T for AF, and postoperative outcome.

**References**	**Type of study**	**No. of pts**	**Results**
Goradia et al. (65)	Pre-operative planning and post-operative ouctome	10	Children undergoing left temporal lobectomy for epilepsy. Increase of fiber density in the right AF
Bizzi et al. (29)	Pre-operative planning	19	Aphasia in patients with gliomas is mainly due to damage of subcortical networks
Zhao et al. (58)	Pre-operative planning and post-operative ouctome	11	Adjustment of pre-operative DTI with intraoperative MRI allows preservation of AF during surgical resection
Kinoshita et al. (2)	Pre-operative planning and post-operative ouctome	12	Significant relation between preoperative increasing value of the FA of the arcuate fasciculus in the dominant hemisphere and postoperative language recovery in patients with gliomas
Caverzasi et al. (61)	Pre-operative planning and post-operative ouctome	78	Preservation of AF/SLF on post-operative tractography is related with recovery from post-operative aphasia
Ille et al. (67)	Pre-operative planning and post-operative ouctome	10	Lose of AF on post-operative MRI is related with non-fluent aphasia
Incekara et al. (68)	Pre-operative planning	77	Significant correlation between FA alterations in AF and language deficits in patients with gliomas
Jehna et al. (19)	Pre-operative planning	27	Language functions at presentation in patients with left hemisphere gliomas were worse in case the AF was left-lateralized
Li et al. (59)	Pre-operative planning and post-operative ouctome	54	Post-operative aphasia is developed when glioma resection reaches <5 mm from AF
Jiao et al. (16)	Pre-operative planning and post-operative ouctome	6	Patients undergoing resection of artero-venous malformations in the left IPL recovered from post-operative aphasia due to a compensatory increase of AF fiber density in the right hemisphere

From a functional point of view, the relevance of asymmetrical DTI-T representation of AF for lateralization of language functions has been partially questioned by studies on left-handers (71, 72). These studies stem from the assumption that the dominant hemisphere for language is also usually associated with contralateral handedness. In a particularly large study, Allendrofer et al. who performed DTI-T in 82 atypical-handers and 158 right-handers, found leftward asymmetry of AF reconstruction in both groups (72). However, in another study, Vernooij et al. (71) showed that, despite the proportion of cases with a leftward asymmetry of AF, reconstruction on DTI-T in left-handers was similar to that of right-handers; when language dominance was also taken into account as revealed by fMRI, the proportion of AF leftward asymmetry in left-handers with right-hemisphere language dominance increased to 100% (72). Thus, even if the degree of lateralization of DTI-T reconstruction alone may not be a good index for predicting language recovery in case of brain damage, it may add valuable information if combined with other functional measure such as fMRI. A method to directly link functional and tractography data is the use of transcranial magnetic stimulation (TMS) combined with coregistration to MRI structural and functional data by means of a neuronavigation software (14, 67). TMS allows a direct cortical and subcortical stimulation from the scalp and is able to induce a transient neurological deficit. Neuronavigated TMS (nTMS) allows to aim the neurophysiological stimulation according to the brain imaging to explore functional responses induced in specific cortical regions (41, 73). In this view, nTMS represents a very useful tool for preoperative neurophysiological mapping of the brain. Many studies show that nTMS has a good correlation with intraoperative findings obtained with direct cortical stimulation (DCS) (74, 75), although a first experience published by Picht et al. about correlation between DCS during awake craniotomy and presurgical nTMS showed a low positive predictive value of nTMS compared to DCS (76). For this reason, as suggested by some authors, nTMS for language mapping should be used with awake DCS, when possible, while nTMS alone should be only used as a rescue measure in patients not eligible for awake surgery with good results (72, 73, 77). In this view, Krieg et al. reported a standardized protocol to reduce technical limitations and increase the accuracy of nTMS language mapping (78). Recently, Sollmann et al. stratified the risk for language deficits using nTMS and described the “lesion-to-tract distance” as a predictive marker of postoperative deficit (74). Thus, combination of nTMS, fMRI, and DTI-T may shed new light on the functional importance of DTI-T asymmetries of AF for language lateralization both for assessing presurgical risk of aphasia and for understanding their roles in functional recovery in case of language deficits induced by surgery.

## Conclusions

Based on our review, subcortical plasticity may play a significant role in compensating the damage to language brain networks induced by gliomas of the language-dominant hemisphere. However, further studies are needed to fully understand its role and how to take it into account for presurgical risk assessment. Studying the whole WM organization, extending DTI-T reconstruction to association bundles connecting homologous language brain areas of the contralesionally hemisphere might be of help in better understanding postsurgical outcome and possible compensatory mechanisms in case of damage of associative WM bundles critical for language and located near the tumor. Moreover, a longitudinal follow-up might be of help in understanding the potential plasticity of subcortical networks after surgery. Navigated TMS could represent the future tool for studying the functional connectivity in brain tumor patients and may be able to better define if and how a right AF could supply to a language deficit induced by damage of a left-dominant AF after surgery. Along with the AF, possible compensatory roles for other long WM bundles in the non-dominant hemisphere, like the ILF or IFOF, might be matter of research with the same methodology and may prepare the path to translate brain connectionist models to the clinical practice.

## Author Contributions

AD, CG, and CdL: concept and writing. GB: writing and elaboration of DTI. MS, IM, CF, and VI: literature analysis and manuscript revision. All authors contributed to the article and approved the submitted version.

## Conflict of Interest

The authors declare that the research was conducted in the absence of any commercial or financial relationships that could be construed as a potential conflict of interest.
